# MTA1 Expression in Benign and Malignant Salivary gland Tumors

**Published:** 2016-01

**Authors:** Azadeh Andisheh-Tadbir, Ali Dehghani-Nazhvani, Mohammad Javad Ashraf, Bijan Khademi, Hosein Mirhadi, Shima Torabi-Ardekani

**Affiliations:** 1 *Prevention of Oral and Dental Disease Research Center, Department of Oral and Maxillofacial Pathology, School of Dentistry, Shiraz University of Medical Sciences, Shiraz, Iran.*; 2 *Department of Oral and Maxillofacial Pathology, School of Dentistry, Shiraz University of Medical Sciences, Shiraz, Iran.*; 3 *Department of Pathology, School of Medicine, Shiraz University of Medical Sciences, Shiraz, Iran.*; 4 *Department of Otorhinolaryngology, Khalili Hospital, Shiraz Institute for Cancer Research, Shiraz University of Medical Sciences, Shiraz, Iran.*; 5 *Department of Endodontics, School of Dentistry, Shiraz University of Medical Sciences, Shiraz, Iran. *

**Keywords:** Adenoid cystic carcinoma, Salivary gland tumor, Mucoepidermoid carcinoma, MTA, Pleomorphic adenoma.

## Abstract

**Introduction:**

Salivary gland tumors (SGTs) are important parts of human neoplasms. The most common SGT is pleomorphic adenoma and the most common malignant SGTs are mucoepidermoid carcinoma and adenoid cystic carcinoma (ACC). Metastasis-associated genes 1 (MTA1), a member of the nucleosome remodeling and histone deacetylation complex, is one newly discovered gene which recruits histone deacetylation, causing ATP-dependent chromosome remodeling, and regulating transcription. MTA1 had been shown to be overexpressed in malignant tumors with the enhancement of invasion and metastasis.

**Materials and Methods:**

Fifty-six samples of salivary gland tumors from the Khalili Hospital archive, including 20 cases of pleomorphic adenoma, 17 cases of mucoepidermoid carcinoma, 19 cases of ACC, and 23 cases of normal salivary gland tissues were chosen for immunohistochemical analysis of MTA1.

**Results:**

MTA1 expression in the malignant tumors was significantly higher than that in pleomorphic adenoma (P<0.001), and higher in pleomorphic adenoma than the normal salivary glands(P< 0.001). In total, 69.6% of normal salivary gland tissues showed MTA1, but all cases of salivary gland tumors were positive for MTA1. High nuclear expression of MTA1 was detected in 83.3% (30/36) of the malignant salivary gland tumors and 45% (9/20) of pleomorphic adenoma, while low MTA1 expression was seen in all of the normal salivary gland tissues. No statistically significant correlation was found between MTA1 protein expression and any clinicopathological features (P>0.05).

**Conclusion:**

Our findings demonstrate that MTA1 was significantly overexpressed in malignant salivary gland neoplasm in comparison to a lower level in benign pleomorphic adenoma, suggesting that MTA1 protein might be involved in carcinogenesis.

## Introduction

Salivary gland tumors (SGTs) comprise 1–4% of all human neoplasms (1). The most common tumor of salivary gland origin is pleomorphic adenoma (PA), with an incidence of 70% (2). Mucoepidermoid carcinoma (MEC) and adenoid cystic carcinoma (ACC), with incidences of 35% and 20%, respectively, are the most common malignant SGTs (3). Because of the various histopathological features of SGTs and their different clinical appearances and morphologies, it is currently not possible to define the prognosis of these tumors (4). 

The molecular mechanisms involved in the development of SGTs are still unclear (4,5). Malignant SGTs are slow growing but ACCs and high-grade MECs behave more aggressively because of perineural invasion, local recurrence, and distant metastasis. To date, there is no definite indicator for the accurate determination of prognosis and tumoral behavior (4). Therefore, it is necessary to find new molecular markers to determine the prognosis and treatment of SGTs (4).

Metastasis-associated genes (MTAs) are a recently discovered family of genes with three subtypes: MTA1, MTA2 and MTA3. MTA1 was first isolated by different complementary DNA screening using the 13762 NF rat mammary adenocarcinoma metastatic cell line (6). 

MTA1 is a component of nucleosome remodeling and histone deacetylation complex (7), with a weight of 80 KDa (8), recruiting histone deacetylation (7), causing ATP-dependent chromosome remodeling, and regulating transcription (7,8). MTA1 controls epithelial-mesenchymal transition (8), which is one of the most important phenomena in invasion and metastasis. 

Some authors have shown that MTA1 overexpression is accompanied by poor prognosis in malignant tumors (9), with the enhancement of invasion and metastasis (10). However, to date there has been no investigation of MTA1 expression in SGTs. Therefore, in this study we attempted to evaluate MTA1 expression in SGTs by using an immunohistochemical approach to establish correlation with tumor grade and stage and to determine prognostic value. 

## Materials and Methods

*I) Patients:*

In this study, 56 samples of salivary gland tumors from the Khalili Hospital archive, Shiraz, Iran were reviewed, including 20 cases of PA, 17 cases of mucoepidermoid carcinoma and 19 cases of ACC. The control group consisted of 23 cases of normal salivary gland tissues adjacent to the previous biopsy of the oral cavity or SGT. Of the 56 patients included in this study, 18 (32.1%) were male and 38 (67.9%) were female. The mean age was 48.36±11.3 (range 7–81) years. Tumor stages were assessed based on the stages adapted by the American Joint Committee on Cancer (AJCC) TNM stage (11). Tumor grade in MEC was classified as grade I if it demonstrated a well-demarcated border, macrocystic spaces and a bland cyst lining; grade II if it demonstrated a more solid growth with only a few microcysts, and focal infiltration; and grade III in the case of no cystic spaces and a highly infiltrative growth pattern, and pronounced nuclear atypia. ACC grade I referred to tubular growth pattern; grade II referred to cribriform growth pattern; and grade III referred to solid growth pattern (12).

*II) Immunohistochemical staining and analysis:*

First, hematoxylin and eosin slides of available blocks were reviewed, and cases with a definite diagnosis and adequate cellular tissue were selected for immunohistochemical staining (IHC). IHC staining was performed by use of an Envision-labelled peroxidase system (DAKO, Carpinteria, CA, USA). All samples were fixed in 10% buffered formalin and embedded in paraffin. Sections of 5-μm thickness were prepared and deparaffinized in histo-grade xylene, rehydrated in graded alcohol and washed with distilled water. Antigen retrieval was performed using DAKO estimation and a target retrieval solution with pH=9, for 20 minutes. Internal peroxidase activity was inhibited by 3% H_2_O_2_. 

Tissue sections were then incubated for 60 minutes with the anti-MTA1 monoclonal antibody (mouse, Abcam Corporation, ab 84136, UK) at 1/400 dilution. 

Brown nuclear or cytoplasmic staining for MTA1 was considered as positive. Omission of the primary antibody was employed as a negative control, while oral squamous-cell carcinoma tissue known to overexpress MTA1 protein was used as a positive control for MTA1 protein staining. 

MTA1 immunoreactivity was evaluated using a semiquantitative scoring system for both the percentage of positively stained cancer cells (0: 0–5%, 1: 6–25%, 2: 26–50%, 3: 51–75%, 4: >76%) and the staining intensity (0, negative staining; 1, weak staining; 2, moderate staining; 3, intense staining). 

The total score was the multiplication of the scores of the staining intensity and percentage of positive cells, and was graded as follows: 0,1, 2, 3, 4, 6, 8, 9, or 12. Tumors with a final staining score >6 were defined as having high MTA1 expression, while tumors with scores ≤6 were defined as having low MTA1 expression (13). 

*III) Statistical analysis:*

A Mann-Whitney test, Kruskal Wallis test, Chi-Square test, Spearman´s correlation coefficient test and Fisher’s Exact test were used to compare the result between groups and the relation with clinical-pathological features such as tumor size, metastasis to lymph nodes, distant metastasis, tumor grade and tumor stage. We used the SPSS18 software to statistically analyze the data. AP<0.05 was considered statistically significant in all cases. 

## Results

Of the 56 patients included in this study, 18 (32.1%) were male and 38 (67.9%) were female. The mean age was 48.36±11.3 (range 7–81) years; patients with PA were the youngest (mean, 42.4±16.2 years) and patients with MEC were the oldest (mean, 57.7±14.4 years). Most tumors (60.7%) involved the major salivary glands.

MTA1 expression in normal salivary gland and salivary gland tumor:

Both cytoplasmic and nuclear patterns were seen in both normal salivary gland tissue and SGTs. In the normal salivary gland tissue, MTA1 staining was positive in 16 cases (69.6%). 

MTA1 expression was seen in the epithelial lining of the salivary duct and also in mucous and serous acini (Fig.1).

**BFig 1:**
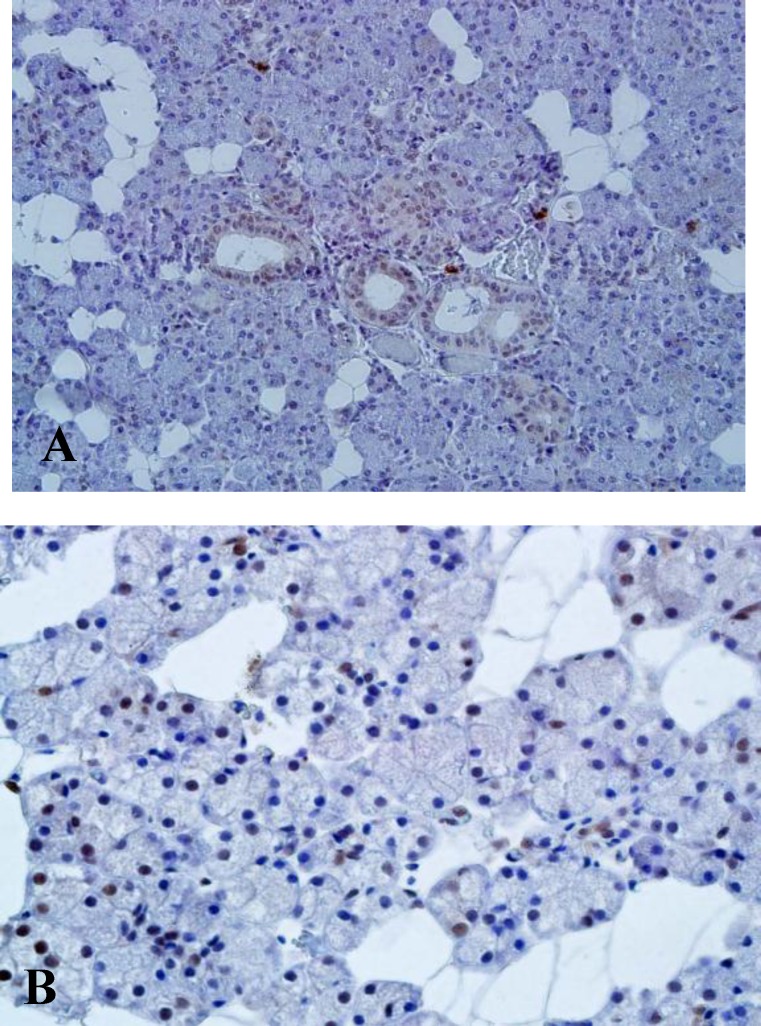
MTA-1 expression in duct and acini of normal salivary gland tissues (A: ×200) (B: ×400

**Fig2:**
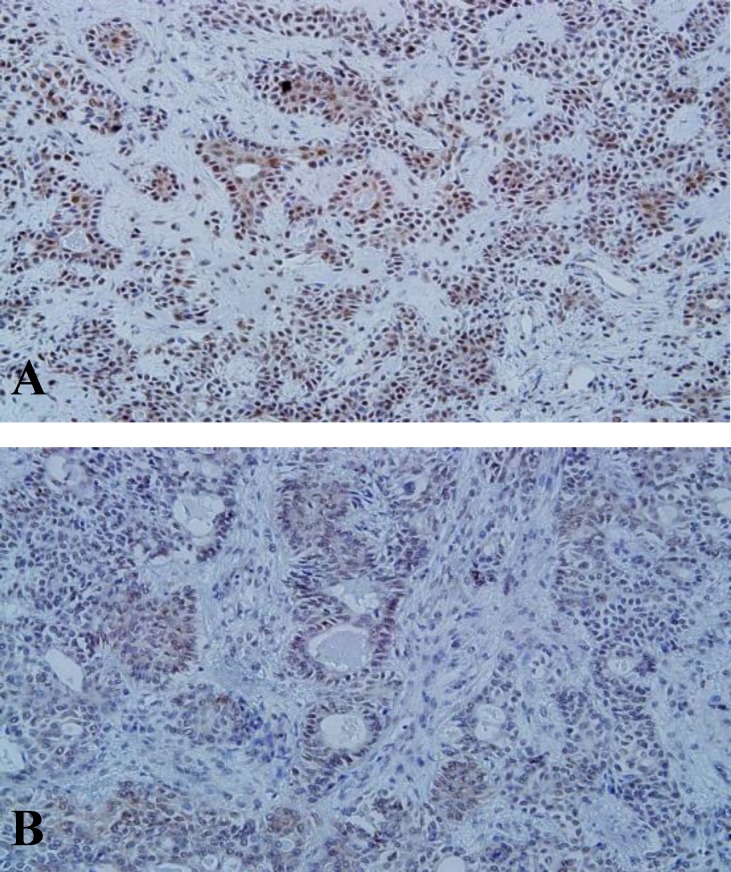
MTA-1 expression in ductal and myoepithelial cells of pleomorphic adenoma (×200

MTA1 immunoreactivity was seen in all (100%) the SGTs. In PA, both ductal and myoepithelial cells showed MTA1 staining (Fig. 2).

**Fig 3:**
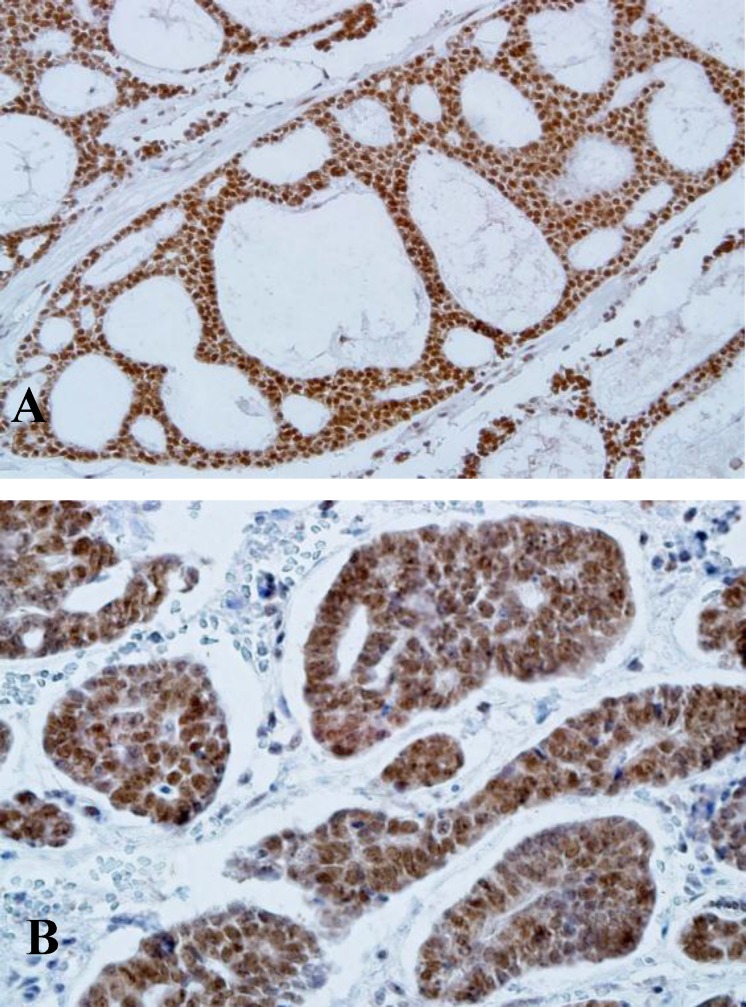
MTA-1 expression in ACC (A:×200)(B: ×400

All histological variants of ACC showed MTA1 positivity (Fig.3).

**Fig 4:**
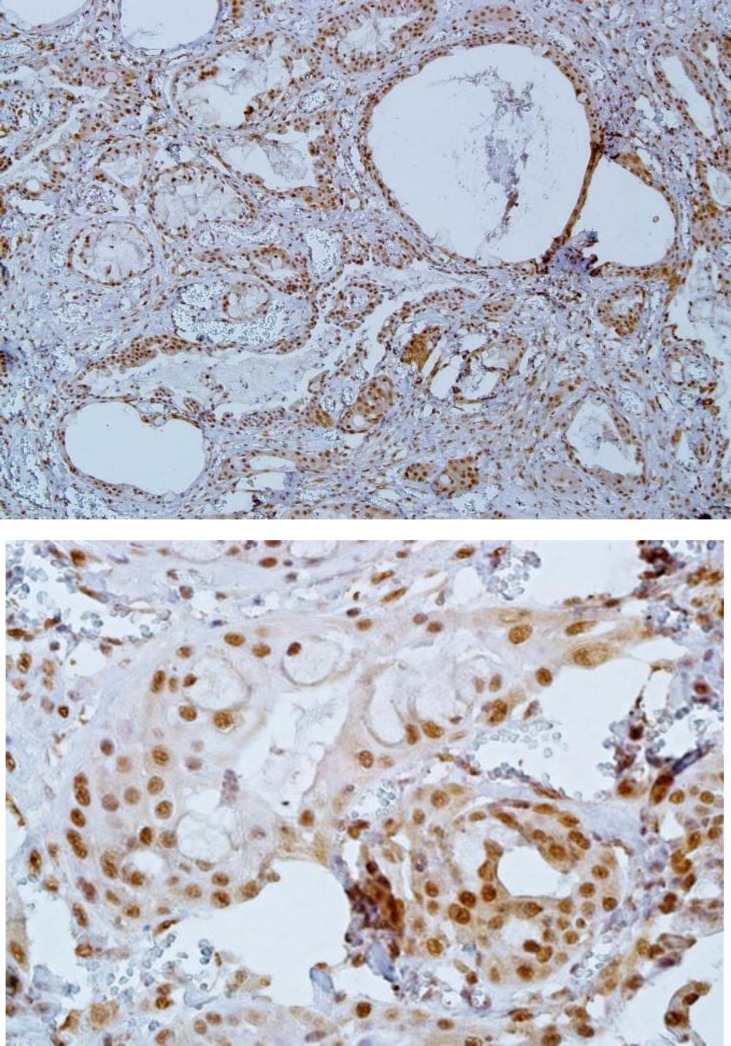
MTA-1 expression in mucoepidermoid carcinoma (A: ×100) (B: ×400

In MEC, the epidermoid cells, mucous cells, and intermediate cells displayed positive staining. Solid areas with predominant epidermoid cells revealed a high expression (Fig.4). 

High nuclear expression of MTA1 was detected in 83.3% (30/36) of the malignant SGTs and 45% (9/20) of PA, while low MTA1 expression was seen in all of the normal salivary gland tissues (Table.1).

**Table:** 

**Types of lesion**	**Number of patients**	**High MTA1 expression** **N (%)**	**Low MTA1 expression** **N (%)**
ACC	19	17 (89.5)	2(10.5)
MEC	17	13(76.5)	4(23.5)
PA	20	9 (45)	11 (55)
Normal	23	0 (0)	23 (100)
			

 MTA1 expression in the malignant tumors was significantly higher than that in PA (P<0.001), and it was higher in PA than in the normal salivary glands (P<0.001). In ACC, 89.5% (17/19) cases showed high nuclear expression of MTA1, while in MEC, 76.5% (13/17) showed high MTA1 expression. No significant difference was seen between MEC and ACC (P>0.05).

Correlation between MTA1 expression and the clinicopathological features of malignant salivary gland tumors:

Our data showed no statistically significant correlation between MTA1 protein expression and any clinicopathological features (P>0.05) (Table. 2). 

**Table 2:** Clinicopathologic characteristics of the 56 patients with salivary gland tumors

**Variable**		**N (%)**	**MTA1 protein** **(high expression, total score >6)N (%)**	**MTA1 protein** **(low expression, total score ≤6)N (%)**	**P. Value**
	Gender				
Male		18 (32.1%)	15 (83.3%)	3 (16.7%)	0.53
female		38 (67.9%)	24 (63.2%)	14 (36.8%)	
Age		48.3 ± 11.3	–	–	0.04
	T-status MEC				
T1+T2		10 (58.8)	7 (70)	3 (30)	0.19
T3+T4		7 (41.2)	6 (85.7)	1 (14.3)	
	ACC				
T1+T2		10 (52.6)	8 (80)	2 (20)	0.4
T3+T4		9 (47.4)	9 (100)	0 (0)	
	N status MEC				
N0		14 (82.3)	10 (71.4)	4 (28.6)	0.5
N1		3 (17.7)	3 (100)	0 (0)	
	ACC				
N0		16 (84.2)	14 (87.5)	2 (12.5)	0.7
N1		3 (15.8)	3 (100)	0 (0)	
	M status MEC				
M0		15 (88.2)	11 (73.3)	4 (26.7)	0.5
M1		2 (11.8)	2 (100)	0 (0)	
	ACC				
M0		18 (94.7)	16 (88.9)	2 (11.1)	0.8
M1		1 (5.3)	1 (100)	0 (0)	
	Stage MEC				
I+II		8 (47.05)	5 (62.5)	3 (37.5)	0.2
III+IV		9 (52.95)	8 (88.9)	1 (11.1)	
	ACC				
I+II		9 (47.4)	7 (77.8)	2 (22.2)	0.2
III+IV		10 (52.6)	10 (100)	0 (0)	
	Grade MEC				
I		7 (41.2)	4 (57.1)	3 (42.9)	0.2
II		10 (58.8)	9 (90)	1 (10)	
	ACC				
I		5 (26.3)	4 (80)	1 (20)	0.3
II		13 (68.4)	12 (92.3)	1 (7.7)	
III		1 (5.3)	1 (100)	0 (0)	

## Discussion

Salivary gland neoplasms are important parts of head and neck lesions (3), but they are rare and comprise only 3–10% of head and neck tumors (14). Salivary gland neoplasms have morphological diversity and wide heterogeneity, so their diagnosis and classification are challenging (15). SGTs with the same histopathological features or stage have different clinical behavior, and therefore, different prognoses (16). Thus, finding new biomarkers that are correlated with tumor prognosis and treatment protocol is necessary. 

MTA1 was primarily isolated by different complementary DNA screening using the rat metastatic breast carcinoma (17). MTA1 is a coregulatory factor and acts in cellular signaling and remodeling of chromosomes, and also has transcriptional activity, so it is involved in progression, growth, and invasion of metastatic epithelial cells (9). MTA1 has been shown to be overexpressed in various human cancers and involved in tumor invasion, higher metastatic potential and advanced clinical stage (18). This study for the first time tried to evaluate MTA1 expression and its prognostic role in SGTs. We found that MTA1 immunoreactivity in malignant tumors was significantly higher than PA and higher in PA than in the normal salivary gland tissues (nSGTs). This finding suggests that MTA1 is involved in the carcinogenesis of SGTs and reflects the aggressive nature of these malignant tumors when compared with PA. These findings are in accordance with the higher levels of MTA1 seen in ovarian cancer in comparison with normal ovarian epithelium (13). 

Some other investigations have also found higher levels of MTA1 in malignant neoplasms such as prostate cancer, gastric cancer, esophageal and renal cancer than in normal tissues (19). The mechanisms of MTA1 function have been studied extensively. MTA1 affects various biomolecules and causes carcinogenesis and tumor progression. P53 is an important tumor-suppressor gene and causes apoptosis. It is deacetylated by MTA1, which results in repression of P53 activity in non-small-cell lung cancer and hepatoma (20). 

Another mechanism of the MTA1 function is in relation to hypoxia-induced factor-1α (HIF-1α). MTA1 increases the expression of histone deacetylase-1 and causes deacetylation of HIF-1α, which is involved in tumor angiogenesis (21). MTA1 can also bind to HIF-1α and deacetylated HIF-1α and cause expression of VEGF-A and VEGF-C, which is associated with metastasis (21,22) and lymphangiogenesis, respectively (23,24). MTA1 is a marker of epithelial-mesenchymal transitions (EMTs). EMTs are necessary for embryonic development and cancer progression (25). 

As discussed above, MTA1 can also be expressed in normal tissue at lower levels. MTA1 at a physiological level may regulate differentiation and EMT (17). However, further studies are required to clarify the exact role of MTA1 in normal tissues. 

Kawasaki found higher MTA1 levels in oral SCC, which was correlated with more advanced stage and higher rates of metastasis to lymph nodes (26). Toh et al. found that a high level of MTA1 protein was associated with depth of tumor invasion, higher pathologic stage, and metastasis to lymph nodes in esophageal SCC (18). Li et al. showed that MTA1 overexpression is correlated with lymph-node metastasis and TNM staging in non-small-cell lung carcinoma, but not with the clinical and histopathological subtypes of tumor (27). In a recent study, the authors showed that MTA1 overexpression in esophageal cancer was correlated with T-status, but no significant correlation was identified with clinical and histopathological status (28).

In the present study, we evaluated the correlation between MTA1 level and clinical-pathological factors such as tumor size, grade, stage and metastasis to lymph nodes. However, we found no correlation between MTA1 overexpression and clinicopathological factors. Although MTA1 overexpression was seen in patients with advanced tumor size and clinical stage, the difference between groups was not statistically significant, which may be because of the low number of cases in our study. Moon et al. reported nuclear and cytoplasmic localization of MTA1 in human hepatocellular carcinoma (HCC) (29). Balasenthil et al. also found both nuclear and cytoplasmic staining for MTA1 in benign endometrial glands, but in endometrial carcinoma they showed that MTA1 was predominantly localized to cytoplasm in grade III tumor, suggesting that in this grade, MTA1 had a non-genomic function (30). MTA1 was shown to have markedly nuclear positivity in some cancerous tissue, such as lung cancer, gastric, colorectal and ovarian cancer (31). In our previous study, we demonstrated both nuclear and cytoplasmic localization of MTA1 in oral SCC (32). 

In the present study, we found both nuclear and cytoplasmic expression of MTA1 in SGTs and nSGTs. Earlier investigations stated that MTA1s, a short version of MTA1, is located in the cytoplasm, binding to ER-α and inhibiting its nuclear function by transforming it to cytoplasm in breast cancer cells (33); thus, non-genomic function of ER-α occurs in the cytoplasm, such as stimulation of MAPK (33). 

Li et al. found cytoplasmic expression of MTA1 in normal adult hepatocytes of the mouse, but after hepatectomy, its expression was predominantly nuclear. This may have been because of the translocation of MTA1 from the cytoplasm to the nucleus to play a transcriptional role and in stimulation of proliferation for hepatic regeneration (34). 

Li et al. (2008) evaluated MTA1 in the salivary gland in a mouse model and found nuclear localization with weak staining intensity in the serous glands, but no staining in the mucous glands, myoepithelial cells or ductal epithelium (35). However, we found both cytoplasmic and nucleus staining in the ductal epithelium and in serous and mucous glands. Predominantly cytoplasmic expression of MTA1 in the serous and mucous acini of the nSGTs in this study may reflect the non-genomic function of MTA1. 

MTA1, master coregulatory biomarker, could be a novel target for cancer therapy, helping to determine the diagnosis and prognosis of tumors (7). The clinical efficiency of targeting MTA1 biomolecules has not yet been proven, but several studies have suggested MTA1 protein or gene as a molecular target for cancer therapy (7). MTA1 has also been shown to stimulate T-cell response in patients with cancer (36). 

Toh et al. also showed that restraining of MTA1 function or expression augmented the sensitivity of cancer cells to chemotherapy by retrieving function of P53 as a tumor-suppressor gene or by preventing tumor angiogenesis by stabilizing HIF-1α (7). Therefore MTA1 is a good target for diagnosis, prognosis, and therapy of human cancers; but more studies are needed to intensively evaluate its role in cancer therapy and its possible clinical use. 

Our study had some limitations, such as an inadequate number of total cases, lack of access to follow up and survival of the patients, and the small number of cases with metastasis to the lymph nodes or distant metastasis. We were, therefore, unable to evaluate the correlation between MTA1 protein level and metastasis. 

## Conclusion

The present study is the first to evaluate the role of MTA1 in benign and malignant salivary gland neoplasms and compare them with nSGTs. Our findings demonstrate that MTA1 was significantly overexpressed in malignant salivary gland neoplasm in comparison with a lower levels in benign PA, suggesting that MTA1 protein might be involved in carcinogenesis; however, further studies are necessary to evaluate MTA1 levels in salivary gland neoplasms and establish its role in diagnosis, prognosis, and cancer therapy. 
